# Computational Approaches Reveal Developmental Shifts in Exploratory Play

**DOI:** 10.1111/desc.70081

**Published:** 2025-10-25

**Authors:** Joseph Colantonio, Ilona Bass, Yee Lee Shing, Sobanawartiny Wijeakumar, Courtney McKay, Eva Rafetseder, Allyson P. Mackey, Elizabeth Bonawitz

**Affiliations:** ^1^ Graduate School of Education Harvard University Cambridge Massachusetts USA; ^2^ Department of Psychology Harvard University Cambridge Massachusetts USA; ^3^ Department of Psychology Goethe University Frankfurt Frankfurt Germany; ^4^ School of Psychology University of Nottingham Nottingham UK; ^5^ Division of Psychology University of Stirling Stirling UK; ^6^ Department of Psychology University of Pennsylvania Philadelphia Pennsylvania USA

**Keywords:** cognitive development, exploratory play, Markov modeling

## Abstract

Although exploratory play is considered a hallmark of cognitive development and learning, relatively few studies have been able to quantitatively characterize the shifts that may occur in children's approach to exploration. One reason for this gap is due to challenges coding and analyzing children's exploratory play behavior. In our paper, we employ a novel computational modeling approach to understand whether and how children's exploratory play patterns shift in early childhood (3‐ to 11‐years‐old). We analyze data from children (*N* = 432) across five different experiments that varied in the type of exploration task (including novel toys, novel topics, and novel envelopes). Children's behaviors were coded action‐by‐action according to whether children repeated an action on the same type of target, switched to a novel target, or terminated play. Our computational Markov model searches over the space of possible “stay,” “switch,” and “end” parameters to quantify child‐specific transition probabilities. We find that overall, older children are less likely to perseverate, more likely to switch, and more likely to end the task earlier. Our approach provides a demonstration of how Markov models can be used to map the process of play, providing insight into theories of developmental changes in exploration.

**Summary:**

- We use Markov models to quantify developmental shifts in children's exploratory play across five naturalistic tasks.
- Older children showed increased exploratory variability and decreased perseveration during play.
- Developmental effects were most consistent in novel toy tasks, but varied across contexts.
- Our findings help reconcile conflicting prior research by highlighting the role of task structure and developmental changes in exploratory strategy.

## 1 Introduction

Consider a child exploring a novel toy with many features—what factors shape the child's choice to persist on a function, to shift and test another, or to decide to stop playing all together? Children's exploratory play supports their learning and so has been a topic of great interest in cognitive development (e.g., Schulz 2012; Singer et al. 2006). Despite the perceived importance of exploratory play in theories of cognitive development, relatively few studies have been able to quantitatively characterize how children's approach to exploration shifts at larger scales—both in the moment as children make decisions, and broadly over the course of development. One reason for this limitation is that quantitative tools are needed to interpret behaviors action‐by‐action and large datasets that contrast relevant factors of the task sessions are hard to come by. In this paper, we explore how a particular computational technique—Markov Chain decision models—can be deployed on large datasets of children's exploratory play, to understand potential developmental shifts in exploration as well as other task‐specific factors that may contribute to children's exploratory behaviors.

### 1.1 The Development of Exploration Strategies in Early Childhood

There has been a growing theoretical debate regarding the development of exploration strategies in early childhood. On the one hand, some have argued that hypothesis search and exploration is broader and more variable in younger children, both in terms of how learners search through hypothesis spaces and in terms of how learners explore the external world (e.g., Gopnik 2020). On the other hand, research has also revealed empirical findings that *older* children demonstrate broader exploration, (more variability or more complexity), with younger children demonstrating more perseverative strategies when exploring the physical world (e.g., Pelz and Kidd 2020).

Scientists advocating for early breadth have investigated children's search in external, physical space as well as their search in internal, mental space. Work from Meder et al. (2021) has suggested that random exploration decreases with age (4‐ to 9‐years‐old), as evidenced by explore‐exploit tasks in tangible environments. In these studies, children were shown a grid with spatially correlated (but unobserved) rewards, which were revealed when clicked. Children were given many trials in which they could choose to click on an already observed space or explore a new one with the goal of maximizing reward. Meder et al. modeled children's behavior to ascertain whether exploration strategies were more random or directed; they found that random exploration decreased with age. That is, younger children were more broadly exploratory in the sense that they entertained more random or “noisy” options than older children and adults, and overall were less efficient at exploiting the task‐structure to maximize reward.

Related work also suggests that in explore‐exploit tasks, younger children tend to make noisier choices (selecting lower‐value options and switching responses more trial by trial), as well as being more likely to trade information gain for reward (e.g., Blanco and Sloutsky 2019; Sumner et al. 2019; see Nussenbaum and Hartley (2019) for review). One potential limitation to this work, as taken as a characterization of children's exploratory play more broadly, is that the task goals (to maximize reward) and the task structure (a sparse grid world, N‐armed bandit tasks) do not look like typical exploratory play in natural settings. This leaves open the question of whether and how exploratory play shifts with development in natural contexts.

There is also evidence for more exploratory breadth earlier in development in tasks that involve an aspect of search in mental space. For example, children have been shown to be better causal learners than adults when the correct causal rule is one that adults typically find a priori unlikely (Lucas et al. 2014; Gopnik et al. 2015, 2017). Younger learners are also more likely to take exploratory “risks” to test (and thus discover) “unlikely” causal rules (Liquin and Gopnik 2022). Furthermore, the guesses younger children generate in hypothesis‐search tasks show greater independence between hypotheses (e.g., bigger “jumps” in terms of semantic similarity) as compared to adults’ guesses which are more dependent (Bonawitz et al. in review). Results like these have supported the claim that the mental search of very young children reflects high exploratory variability, with a “cooling off” or narrowing of search proposals as children develop (Gopnik 2020).

Taken together, the results above suggest that younger children's behavior is marked by broader exploration—both in terms of choices to explore the physical world, and in terms of the hypotheses entertained. However, these tasks were designed to test specific behaviors in explore‐exploit and causal learning paradigms and do not reflect more natural exploratory play tasks. As such, the particular factors of the designs (such as maximizing reward goals, searching for a specific causal rule, etc.) may not reflect the kinds of goals or approaches that children deploy in more natural exploratory play settings. Thus, while this work provides rich theoretical insights into the development of cognitive mechanisms that may support learning and search in early childhood, the results may not apply more broadly to the kinds of search behaviors that children take during more natural exploratory play tasks, such as engaging with toys or digital game environments.

In contrast to the early breadth accounts, other work has suggested that variability may increase with age. For example, in a mega‐analysis of preschoolers’ play behaviors on physical novel toys, Bass and Bonawitz (2024) found that variability (coded in terms of total number of different features attempted) increased between the ages of 4–6‐years‐old. This work did not involve coding of more diverse behaviors, including perseveration or transitions, but provides one piece of evidence that in the preschool years, developmental shifts in play behavior may be emerging.

Similarly, Pelz and Kidd (2020) found that younger children's exploration in naturalistic play tasks is less broad and more perseverative than older children's. These tasks involved giving children (ages 2–12‐years‐old) a Novel Tablet task, where they were testing out which kinds of food a monster might eat. Children explored a touchscreen tablet app called Toca Kitchen Monsters, developed by Stockholm‐based Toca Boca in this task. Pelz and Kidd coded the complexity (breadth and non‐repetitive exploratory play patterns, residualized by total number of touches) of children's choices and found greater complexity scores with age. These results suggest, in contrast with the data above, that younger children show more perseveration and the breadth of children's exploration *increases* with age.

Although Pelz and Kidd (2020) present an ambitious and rich study of the development of children's exploration in naturalistic settings, one limitation of this work is that it has only been examined in the context of one specific play task. This limits the generalizability of these findings to some extent, especially in the context of the broader literature suggesting children's exploratory variability decreases with age. Could developmental effects be related to particulars of this one play task? If so, what are those particulars? How robust might the developmental effects be across other exploratory play paradigms?

Furthermore, although Pelz and Kidd deployed an ingenious computational method to model these data (using a compression tool to measure “complexity” of children's play choices as a function of file size), this tool limits the degree to which we can explain this complexity result. This limitation exists because emerging complexity in exploratory play, as generated by the compression tool, could be driven by several independent or overlapping factors. If younger children are more perseverative, the compression score would be smaller and complexity would thus increase with age. If older children are more variable explorers than younger children (i.e., trying more options, switching between options more quickly, or showing more variability in the amount of time spent with each item), the compression (and thus complexity) score would also be smaller for younger children and increase with age. Finally, longer exploration patterns in older children would additionally lead to greater complexity scores with age.^1^ Pelz and Kidd suggested that the changes in behavioral complexity observed across development was driven by more efficient search in older children. We suspect this is true, but new approaches would be needed to isolate these different components of exploratory play to demonstrate whether (a) younger children are more perseverative, (b) older children are more exploratory, and/or (c) older children resolve play *earlier* than younger children in naturalistic exploratory play environments.

Relying on a single exploratory play paradigm may limit our understanding of how these effects manifest across different contexts. The apparent conflict regarding whether exploratory variability increases or decreases with age reinforces this limitation. This highlights the need for examining multiple tasks, to properly assess the generalizability of developmental effects. By combining studies that employ various play tasks, we can determine whether the observed developmental patterns hold consistently or are task‐specific.

However, there exists a significant challenge to modeling large‐scale and varied play results. What computational approach can be applied across a diverse set of tasks? Computational models, and the parameterizations that shape their interpretation, are typically task‐specific, limiting the degree to which they can be deployed generally across different task structures. Pelz and Kidd's compression approach could provide one promising start, but as noted above, potentially confound our understanding of variability and perseveration into a single complexity score. To better understand exactly which factors are changing with development, we need tools that differentiate between perseveration, breadth, and completion.

### 1.2 Approach

A major goal of the current work is to leverage computational modeling methods to better understand the interactions of development and context during naturalistic play. To answer them, we will employ a computational modeling approach, extending well‐known methods in decision making—Markov Chain decision models (Sutton and Barto 1998; Bellemare et al. 2016; Brändle et al. 2022)—and applying them toward the goal of better understanding exploration as a naturalistic behavior that is possibly more complex and less goal‐bounded compared to more popular operationalizations of exploration (Brändle et al. 2022; Rule et al. 2024). Past work has used similar approaches to examine the shifting strategies that children use in exploration as objective, computational representations of their “action‐by‐action” decisions. For example, Hidden Markov Models have been applied to developmental data to reveal transitions between unobservable strategies that guide children's behaviors (e.g., Blakey et al. 2016; van Bers et al. 2011; Lichtenberg et al. 2023; Visser 2011). These methods are notable for identifying such “hidden” latent shifts in strategy by inferring changes from action sequences (see also Visser and Speekenbrink 2010). In the current work, we deploy this computational step instead to capture individual children's behaviors in terms of whether they “stay” on their current exploratory target, if they “switch” to another point of focus, or if they decide to “end” their play session entirely.

In addition, we examine multiple tasks to better assess the generalizability of developmental effects. However, modeling large‐scale and varied play data presents a significant challenge, as many models are tailored to specific task features, limiting their generalization across contexts. By extending the use of Markov Chain models to represent children's exploration across different tasks, we provide a unified framework for comparing exploratory changes in development. This modeling approach allows us to quantify key components of behavior—such as perseveration, breadth, and termination—across multiple tasks, that may differentially change with development.

### 1.3 Predictions

In the current work, our goal is to better understand children's exploratory play in naturalistic environments. We will specifically focus on the developmental and contextual factors that may shape their play and model the data from children across five different experimental studies that vary in their exploratory play task: including novel toys, novel topics, and novel envelopes (*n* = 432 unique children). Each experiment presents a unique context for children's exploratory play and information seeking in naturalistic ways that they may typically interact with early in life—such as hands‐on, physical toys or within online, digital game environments. Importantly, each task recorded children's “action‐by‐action” instances of exploration, allowing us to examine potential developmental shifts of perseveration, switching, and ending separately.

By combining the five larger datasets and novel modeling approaches, we are able to explore hypotheses about the development of children's exploratory play across different types of task structure. We will analyze children's (3‐ to 11‐years‐old) exploration during play to better understand the described developmental trajectories. Here, we consider two broader hypotheses regarding possible main effects of age. One hypothesis suggests that if children's exploratory play follows explore‐exploit tendencies with “cooling off” over the course of development (e.g., Gopnik 2020), then we may find that older children are less exploratory, as evidenced by higher perseveration scores and lower switching scores in the model. In contrast, an alternative hypothesis may be that children's exploration instead becomes more “variable” with age (e.g., Pelz and Kidd 2020) and we may instead see children move toward broader exploratory choices later in development. We can also explore the relative trade‐offs of these scores with respect to how soon children choose to end the exploratory play task, to assess whether staying and switching scores are consistent controlling for how long children choose to engage with the toy. Finally, we also explore these results in the context of specific tasks, to better understand the degree to which these behaviors are global or may vary depending on specifics of task type.

## 2 Methods

In the current work, children's play data were only included if their participation met the inclusion criteria of the original study for which their data was collected. The main findings of each study are included in their original publication(s), if in review or if published, and will otherwise not be discussed at length in the current manuscript unless relevant to the current computational investigation. Across the five studies, this yields sequential exploratory play data from 432 children (ages 3‐ to 11‐years‐old; *M_age_* = 5.99 years). Participants lived either in the United States (Studies 1, 3, 4, 5) or the United Kingdom (Study 2) at the time of testing. See Table 1 for side‐by‐side comparisons of the design features that varied across the five studies.

**TABLE 1:** Details on the experimental design for each of the five studies. Each of the five studies varied in a number of ways. In the current work, we focus on the specific features of each study and declare them when relevant, including mention of the specific task administered to measure children's exploration (Themed Envelopes, Novel Toys, or Science facts) and the age range of the participants (*n *= 432 unique children; 3‐ to 11‐years‐old; *M_age _* = 5.99 years). We also report but do not directly investigate additional information regarding the experiment's comparison design (within‐ or between‐), details on the exploratory play affordances, and whether play was measured using physical toys or digital stimuli. The asterisk (*) for Study 2's “Between” for subject design notes that some children (*n* = 16) completed the task at multiple time points, due to the longitudinal nature of Study 2.

	Theme envelopes (Study 1, *n* = 83)	Theme envelopes (Study 2, *n* = 64)	Novel toys (2) (Study 3, *n *= 114)	Novel toys (2) (Study 4, *n* = 100)	Science facts (Study 5, *n* = 71)
Age	5‐ to 8‐years‐old *M_age_* = 6.31 years	5‐ to 8‐years‐old *M_age_* = 6.77 years	3‐ to 8‐years‐old *M_age_* = 5.43 years	3‐ to 8‐years‐old *M_age_* = 5.59 years	5‐ to 11‐years‐old *M_age_* = 7.66 years
Subject design	Between	Between*	Within	Within	Between
Exploratory affordances	16 envelopes available for exploration of “new” information; play otherwise “exploits” or returns to previous discoveries (opened envelopes)	16 envelopes available for exploration of “new” information; play otherwise “exploits” or returns to previous discoveries (opened envelopes)	Only functions, some perceptually distinct but inert parts, and a general “blank/inert” click were coded	Functions, multiple distinct inert regions, and a “blank/inert” region of background click were coded	9 topics with 5 facts each, returning to a previous topic is possible but facts were one‐time only.
Stimuli type	Physical	Physical	Digital	Digital	Digital

All data preparation, handling, and analyses are pre‐registered and publicly available (masked for peer review; https://osf.io/agyju/?view_only=62c27a0372474e93a0f5a7c05ceaafb5) via the Open Science Framework. This paper focuses on the first and primary question 1 in the preregistration (developmental effects on overall scores). Exploratory analyses that were performed post hoc are noted as such below.

### 2.1 Study Descriptions

#### 2.1.1 Studies 1 and 2: Themed Toy Envelopes

The play data from Study 1 (*n* = 83; ages 5‐ to 8‐years‐old; *M_age_* = 6.31 years) were collected for a study reported in Park et al. (in review). Park et al. (in review) measured children's exploratory play on the last day of a 10‐day teaching intervention, designed to bolster curiosity and increase science knowledge learning through science lessons. The play data from Study 2 (*n* = 64 children; ages 3‐ to 8‐years old; *M_age_* = 6.77 years) were collected for a currently unpublished study. Study 2 had a longitudinal design, where some (*n *= 16) children completed play sessions at two time points, approximately 1 year apart, and was conducted to investigate the effects of experience with formal schooling on a variety of facets of cognitive development.

A schematic overview of the layout of the themed envelope exploration task can be found in Figure S1A. Children participated in the envelopes task remotely while at home, over Zoom or Microsoft Teams with a live experimenter. The child's parents received a package in the mail containing a short instruction sheet for the parent and 16 equally‐sized envelopes (four sets of four different colors). Each of the envelopes contained a small toy based on the theme of the color set (e.g., the blue envelopes in Study 1 contained toys related to principles of magnetism that interacted with each other). Following a pedagogical question from the experimenter (“How might the things in the blue envelopes go together?”) children were allowed to open envelopes and interact with the objects inside for up to 6 min or until spontaneously claiming they were finished and confirming their decision.

Two research assistants coded the “color‐by‐color” sequence that children opened envelopes in, as well as the sequence of play with the item already opened (but labeled according to the original color‐category of the item given its original envelope color). Interrater reliability was assessed via Intraclass Correlation for the number of unique actions performed by each child. There was good reliability in Study 1 (*ICC*(3, 2) = 0.89, 95%CI [0.84–0.93]). Data was collected at two time points in Study 2, with good reliability for time 1 (*ICC*(3, 2) = 0.79, 95%CI [0.65–0.87]) and time 2 (*ICC*(3, 2) = 0.75, 95%CI [0.55, 0.87]). This provided a measure of whether sequential play choices were within color‐category or switching to a new color category.

#### 2.1.2 Studies 3 and 4: Online Novel Toy Simulator

The play data from Study 3 (*n* = 114 children; ages 3‐ to 8‐years old; *M_age_* = 5.43 years) were collected for Bass et al. (2025), as part of a broader multi‐module study (Sheskin et al. 2020). The play data from Study 4 (*n* = 100 children; ages 3‐ to 8‐years old; *M_age_* = 5.59 years) were collected for a currently unpublished study. Both studies were conducted via the platform Children Helping Science (Scott and Schulz 2017). Studies 3 and 4 include within‐subjects designs where children experienced multiple experimental conditions (pedagogical and accidental demonstrations). In our analyses, we treat each child's play sessions separately for each toy as two unique observations, yielding 228 samples in Study 3, and 200 samples in Study 4.

The procedure for the exploratory play portion (see Figure S2A for a schematic overview) of these two Novel Toy studies is nearly identical. Here, children first saw one of two teachers demonstrate a specific function on a virtual novel toy on a computer screen. Based on the condition, this first teacher either demonstrated the function intentionally in the Pedagogical Directives condition (e.g., “This is my toy! I'm going to show you how my toy works …”) or accidentally activated the function in the Accidental condition (e.g., “I've never seen this toy before! I wonder how it works… Oops, did you see that?”). After watching this first video, the child was then allowed to play with the digital toy in the video. The digital toy contained many possible visual affordances that could link to possible outcomes (e.g., semi‐surreptitious buttons that made fans spin, lights turn on, music play, revealed hidden compartments, etc). After confirming they were done playing, or after their allotted time expired, children then saw a second, new teacher's video in the remaining condition before being given the opportunity to play with a second, different novel toy. Measures of learning were collected after both play sessions were completed in each study; we return to these scores in the exploratory analysis.

During both play sessions, children's click‐by‐click behavior (for both active and inert regions of the toys) was automatically recorded to track their exploratory decisions such as activating‐turning off the same functions repeatedly or testing other potential functions. This provided a measure of whether sequential play choices were within color‐category or switching to a new color category.

#### 2.1.3 Study 5: Science Facts

The data in Study 5 (*n* = 71 children; ages 5‐ to 11‐years old; *M_age_* = 7.66 years) are not currently submitted or published. The exploration task in Study 5 was adapted from methods in Evans and Jirout (2023), and conducted online using Qualtrics. See Figure S3A for a screenshot of the exploration space used in Study 5.

In this exploration task, children were presented with a grid of nine shapes that they could click on to learn “cool facts” about different topics. Each shape was matched to its own, specific science topic (e.g., Desserts, Space, Seals), and each topic had five unique facts (e.g., “The creator of cotton candy was a dentist…”). After clicking on a shape, the game switched screens to display an image and play a recording of a cool fact about a topic. After hearing all five of a topic's facts, additional clicks on the topic's shape led to a screen transition with text and narration (“Wow! You got to hear all of the facts that we have about [topic]! If you'd like, you can go back to the previous page and pick another topic to learn about”) before returning children to the shapes grid for additional exploration. Children's free exploration was not timed, and play only ended when they clicked “ALL DONE!” at the bottom of the screen and confirmed their decision on a new page (“Are you all done playing?”) by clicking on the “YES” button on a confirmation page. Clicking “NO” returned the child to the shape grid.

Children's click responses were automatically coded by the computer click responses. Children's exploration was measured by tracking the click‐by‐click transition among the nine available topics, allowing us to measure whether children tended to persevere and explore a single topic deeply, or switched among the different topic options.

### 2.2 Analysis Plans

#### 2.2.1 Data Preparation

In order to perform the broader analysis and include children's play measures across all five studies, we categorized children's “action‐by‐action” behaviors within each study as one of three possible transition states. Children's actions were coded as “stay” when choosing to interact with the same target, “switching” when choosing to explore a different option from the one immediately beforehand, or “ending” their play.

#### 2.2.2 Computational Modeling Approach

**FIGURE 1:**
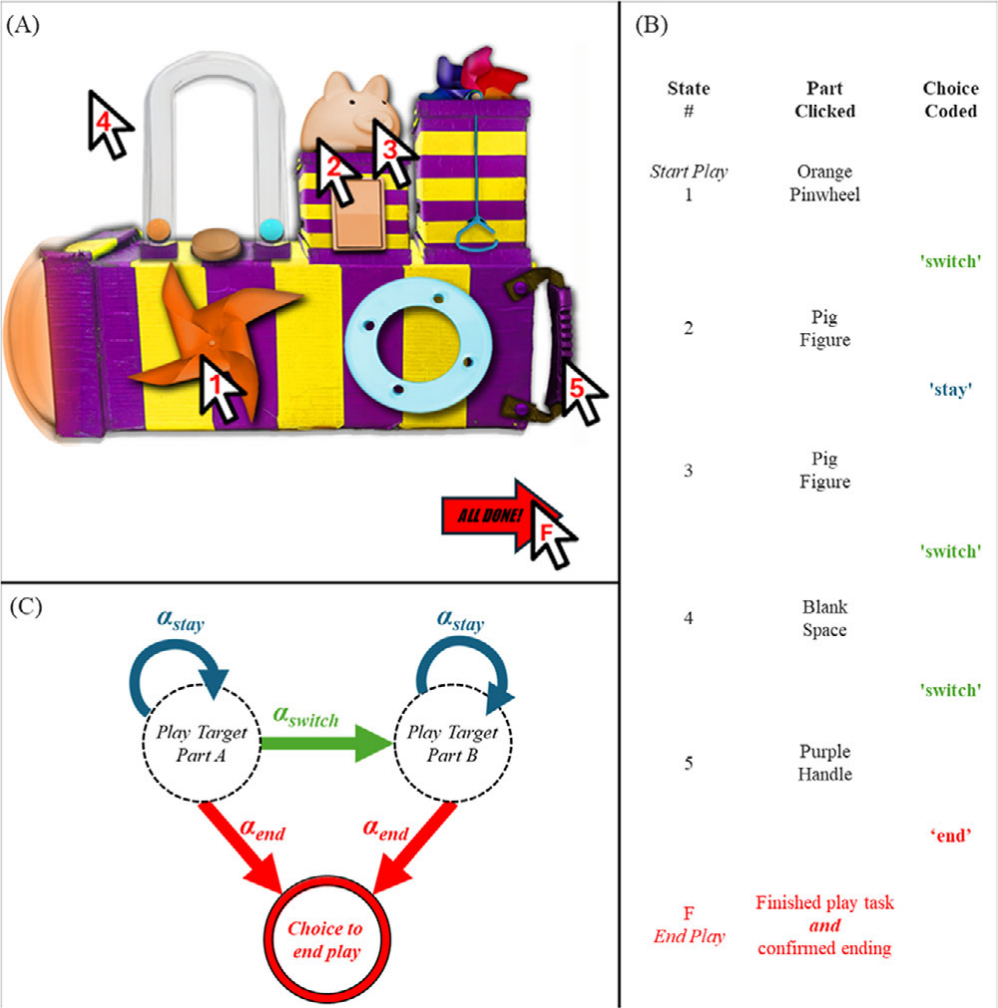
(A) A visual example of one of the toy and task types explored. (B) Example table listing the action‐by‐action sequences of clicks a hypothetical child may have performed. (C) Graphical representation of the Markov decision process employed in the current work. In our computational model, children can either “stay” on their current target of exploration or “switch” to a different target. Decisions to “end” result in termination of play (referred to as an absorbing state in the literature).

Across the five described studies, we employed a Markov Chain model^2^ to estimate three transition indices based on children's play behavior: a “stay,” a “switch,” and an “end” score. Specifically, during their play, children could perform one of three play “transitions.” The indices of “stay,” “switch,” and “end” were taken as transition probabilities between states based on the observed sequence of transitions. See Figure 1 for a visual example of this process using a Novel Toys paradigm (e.g., for Studies 3 and 4), and the Markov Chain decision process employed in the current work. Here, these transition probabilities were calculated directly from the observed frequencies of children's actions, yielding Maximum Likelihood Estimates of the three indices. This means our parameters were deterministically derived as direct calculations from the data, as compared to estimates that may have been obtained through methods based on stochastic or iterative optimization algorithms.

First, we combined the entirety of the available data (*n *= 662 play sessions for 432 unique children across the five studies) and looked at the correlations between each of the three transition indices (“staying,” “switching,” “end”) and children's age. Generally, if children's exploratory play is less variable with age, then we should assume that children's staying scores will positively correlate with age across the studies, and switching scores should negatively correlate with age. In contrast, if children's exploratory variability increases with age, then we should see staying scores negatively correlate with age and switching scores increase with age.

This dataset also lends itself to further analysis of the three different exploratory play task types. This allows us to assess whether specific tasks might differently lead to overall changes in the development of children's play transitions.

To examine how these different study contexts may influence children's play transitions, we employed Dirichlet regression models^3^ (see Maier 2014). This is because traditional regression models typically assume independent outcome variables. Here, the compositional nature of the play transition scores imposes an inherent constraint (as staying, switching, and ending being probabilities must sum to 1), thus necessitating our account for their interdependence rather than treating them as independent quantities. By using Dirichlet regression, we can also capture trade‐offs among different play transitions (if one increases, the others must decrease).

To assess both the direct effects of the study conditions and their interactions with age in shaping the proportional distribution of play transitions, Dirichlet regression models were specified as (*Stay, Switch, End*) ∼ Dirichlet(*α_1_, α_2_, α_3_*).

## 3 Results

The main analyses aimed to test the effects of age on children's exploratory play measured through three possible behaviors they may perform: staying on their current target of exploration, shifting their exploratory focus, and ending their search altogether. We will first look at the correlations between each of the three transition indices (“stay,” “switch,” “end”) and children's age. Then, Dirichlet regression was used to estimate the relationships between these predictors and the estimated transition indices of “stay,” “switch,” and “end.”

### 3.1 Developmental Changes Across All Tasks Combined

We first looked directly at the relations between development and each play score via the correlation between children's age and their individual stay, switch, and end scores. Here, we found significant correlations across all three scores. We found a negative correlation between age and children's stay scores (*r*(660) = –0.184, *p* < 0.001), suggesting greater perseveration in earlier childhood. We found a positive correlation between age and switch scores (*r*(660) = 0.163, *p* < 0.001), suggesting greater exploratory variability in later childhood. Additionally, we found a positive correlation between age and children's end scores (*r*(660) = 0.122, *p* < 0.01), suggesting increasing desires to terminate the exploratory play session earlier with age. These results align with data from Pelz and Kidd (2020), suggesting that older children's play behaviors are more complex with age.

**FIGURE 2:**
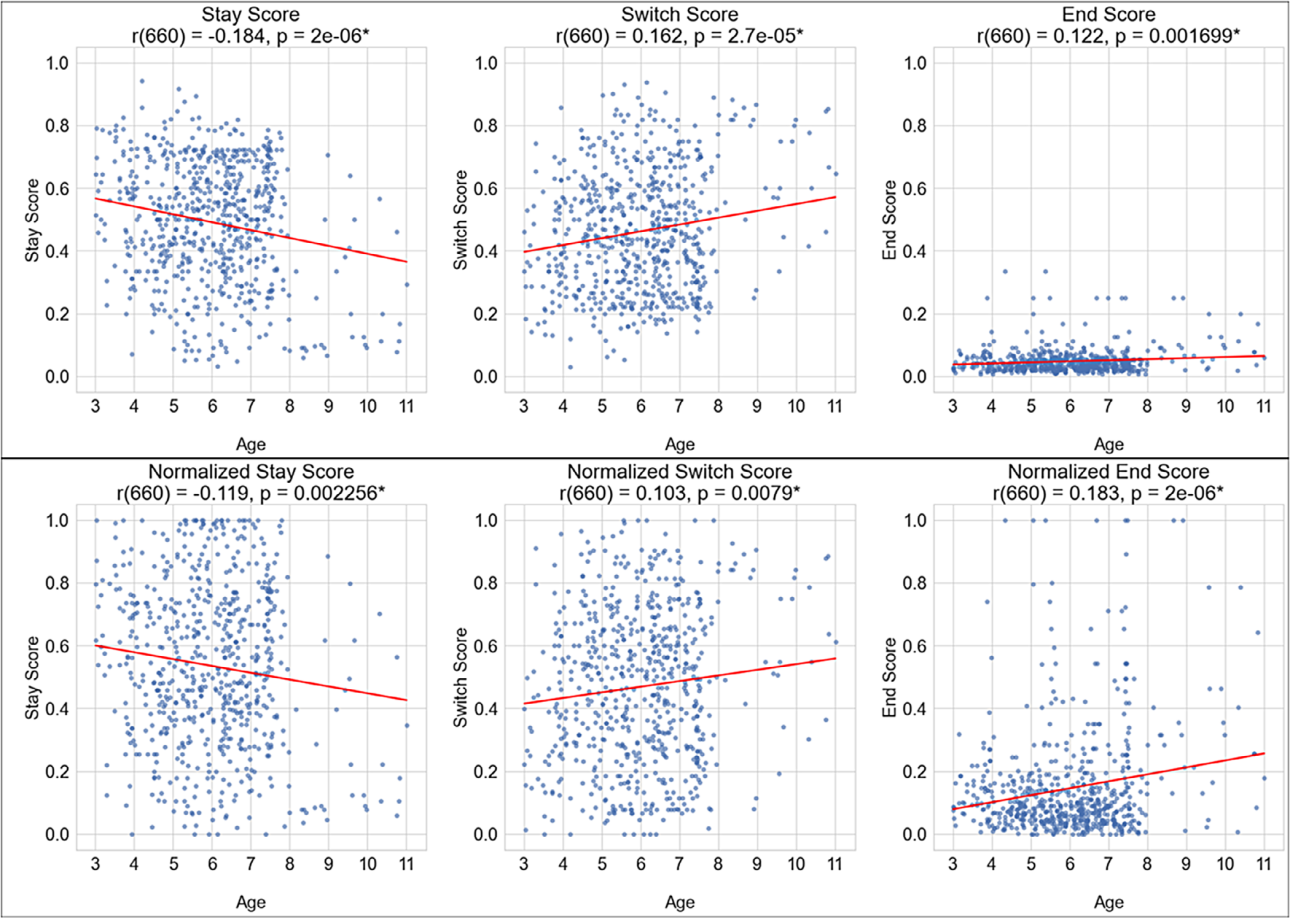
Correlations between children's age and each of their estimated play transition scores (stay, switch, end; Top Row). Correlations between children's age and each of their within‐study min–max normalized play scores (Bottom Row). In both cases, children's age had a significant negative correlation with their stay scores, a significant positive correlation with their switch scores, and a significant positive correlation with their end scores.

To control for possible confounds that may arise from unique task demands across studies (as they were designed based on differences among within‐study conditions), we also calculated children's min–max normalized score (within‐study). The direction and significance of the correlations remained when considering these normalized transition scores, with age having a negative correlation with (normalized) stay scores (*r*(660) = –0.119, *p* < 0.01), a positive correlation between age and (normalized) change scores (*r*(660) = 0.103, *p *< 0.01), and a positive correlation with (normalized) end scores (*r*(660) = 0.183, *p* < 0.001). See Table 2 for details, and Figure 2 for a visualization of the relation between children's three scores (stay, switch, end; as well as the min–max normalized scores) and their age.

**TABLE 2:** Correlations between children's age and each of the three play transition scores across the full dataset (all five studies combined). Significant correlations are noted with and asterisk (*) and bold formatting. The top row lists the correlations between children's age and each of the three play transition scores as calculated by our Markov model. The bottom row lists the correlations instead, using the within‐study, min‐max normalized scores to account for potential within‐study demands. In both treatments of the play transition scores, we found significant effects of development regarding children's exploratory play.

Correlation between Age & Play Transition Score	Stay Score	Switch Score	End Score
	***r*(660) = –0.184** *p *< 0.001*	***r*(660) = 0.162** *p* < 0.001*	***r*(660) = 0.122** *p* < 0.01*
Correlation between Age & Normalized Score	Within‐Study Normalized stay	Within‐Study Normalized switch	Within‐Study Normalized end
	***r*(660) = –0.119** *p *< 0.01*	***r*(660) = 0.162** *p* < 0.01*	***r*(660) = 0.122** *p* < 0.001*

Finally, we performed an exploratory analysis looking at the partial correlations between age and either stay or switch scores, while controlling for end scores. This additional analysis was conducted to highlight how development may influence children's decisions to balance exploring either more deeply or more broadly, as the influence of end scores could be a possible confound due to the compositional nature of our outcome variables (stay, switch, end).^4^ Partial correlations are reported side‐by‐side in Table 3. Here, we first found that even when considering the possible influence of “end,” children's age still had a significant negative correlation with stay (*r*(660) = –0.163, CI 95% [–0.24, –0.09], *p* < 0.001). Furthermore, the significant relation between age and switch scores also remained, with a significant positive correlation (*r*(660) = 0.163, CI 95% [0.09, 0.24], *p* < 0.001). Together, this suggests that the mechanism underlying development of broad exploratory play over perseverative deeper exploration may be complex and is not limited by the time children decide to finish playing.

**TABLE 3:** Exploratory analysis of the partial correlations between children's age and each of their stay and switch play scores, controlling for their end scores, for the full dataset (all five studies combined). Significant correlations are noted with and asterisk (*) and bold formatting. This conservative approach supports our main results and suggests that children's exploratory play may develop from more persistent, deeper dives early in childhood, toward evolving into broader searches during play as they grow older.

Partial correlation Controlling for End Scores	Stay Score	Switch Score
	***r*(662) = –0.163** CI 95% [–0.24, –0.09]* *p* < 0.001*	***r*(662) = 0.163272** CI 95% [0.09, 0.24]* *p* < 0.001*

#### 3.1.1 Dirichlet Regression Models

To test the relationships between age and the relative proportions of play transitions, we ran Dirichlet regression models. This allows us to assess whether the relative weights of types of transitions differ with respect to each other, as a function of age. In the first model, by treating staying as a reference, we can interpret age effects on switching and ending in relation to staying. Positive age effects on a score mean that children exhibit more of that behavior (relative to staying) as they get older; negative effects mean that behavior decreases with age (relative to staying).

*Effects of Age on Exploratory Transitions*. The regression results indicate that age significantly predicts children's play transitions. Specifically, age was positively related to the likelihood of switching to a new play target and spontaneously deciding to end play, rather than staying with the current play target. The coefficient for age on switch scores was (*β* = 0.11436, *SE* = 0.01764, *z* = 6.482, *p* < 0.001), suggesting that older children were more likely to switch their focus compared to younger children. Similarly, age was positively related to end scores (*β* = 0.09389, *SE* = 0.02687, *z* = 3.494, *p* < 0.001), indicating that older children were also more likely to end their play earlier than their younger counterparts. The results of the Wald test indicated that while age had significant effects on both switching and ending, the difference in the magnitude of these effects was not statistically significant (*W* = 0.567, *p* = 0.45).

Because children in Studies 2, 3, and 4 completed two play sessions, we wanted to ensure that our results were not unduly influenced by potential non‐independence of repeated measures within children. To this end, we reconducted additional analyses using just the first play session from each child (*n* = 432 of 662 original observations). We qualitatively replicated our results within this “first‐play” subset, suggesting that our findings are not solely driven by the inclusion of multiple observations from the same individuals; see Supporting Information Appendix, Analysis of Children's First Play Sessions, for additional details.

### 3.2 Effects of Development Within‐Study

**FIGURE 3:**
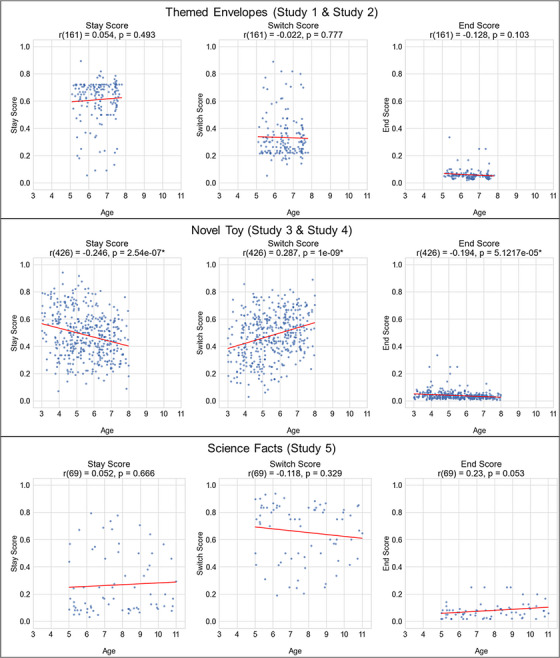
Correlations between children's age and their estimated play transition scores (stay, switch, end) within each task type (exploration of Themed Envelopes, OnlineNovel Toys, and Science Facts). When exploring Novel Toys (Middle Row), there were significant correlations between age and all three play scores.

Our next goal was to examine whether individual task types varied in terms of the developmental effects of exploration variability. For Studies 1 and 2, looking at the Themed Envelopes task, no significant correlation was found (*p* > 0.05 for all play scores). We observed a significant correlation between development and children's transition scores within the two (Studies 3 and 4) digital Novel Toy tasks^5^ (see Figure 3 for visualization of the with‐study relations between age and children's three scores; Table 4 lists all details for within‐study correlation metrics). Looking at children's exploration of Novel Toys (Studies 3 and 4) revealed significant correlations in line with the correlations of the full dataset. The correlation between age and stay scores was negative and significant (*r*(426) = –0.246, *p* < 0.001); Age and switch were significantly, positively correlated (*r*(426) = 0.287, *p* < 0.001). Finally, the age and end score correlation was significant and negative (*r*(426) = –0.194, *p* < 0.001). For Science facts, only a marginal positive correlation between children's age and end scores (*r* = 0.23, *p* = 0.053) was found. Finally, while controlling for end scores, partial correlations between age and both stay and switch scores reveal the same significant trends, with development leading to significantly lower stay scores and significantly higher switch scores only within the Novel Toy play contexts (See Table S1A).

**TABLE 4:** Correlation between age and children's transition scores (stay, switch, end) collapsed within task type (Themed Envelopes, online Novel Toys, or Science facts). Significant correlations are noted with an asterisk (*) and bold formatting. Our main results only held within contexts where children explored Novel Toys (Middle Row, Studies 3 and 4), suggesting that with development, older children were significantly less likely to persist in continuously exploring a single part of the toy, significantly more likely to explore multiple parts of a Novel Toy, and significantly more likely to terminate their playful search early.

Task used	Correlation between Age & Play Transition Score		
	Stay Score	Switch Score	End Score
Themed Envelopes (Study 1 & Study 2)	*r* = 0.054 *p* = 0.493	*r* = –0.022 *p* = 0.777	*r* = –0.128 *p* = 0.103
Novel Toy (Study 3 & Study 4)	***r* =** –**0.246** *p* < 0.001 *	***r* = 0.287** *p* < 0.001*	***r* =** –**0.194** *p* < 0.001*
Science Facts (Study 5)	*r* = 0.052 *p* = 0.666	*r* = ‐0.118 *p* = 0.329	*r* = 0.23 *p* = 0.053

#### 3.2.1 Learning Outcomes

As an exploratory (non‐preregistered) final measure, we looked at the relationship between children's learning outcomes and age. While we do not have learning measures on the “Envelopes” or “Science Facts” tasks, we did have learning outcomes for the Novel Toy tasks. In this task, we measured learning scores for both toys (separately) in the Novel Toy tasks. After completing play with both toys, children were again presented with each toy (one‐at‐a‐time) and asked follow‐up questions about how to activate the four functions of that specific toy (“*Where do you need to click on the toy to make it do this?*”). Each child received a learning score equal to the number of correctly answered questions, ranging from 0 to 4 (for each toy). Combining the data from Studies 3 and 4 (*N *= 214 children), we found that age significantly correlated with children's learning scores (*r*(212) = 0.295, *p* < 0.001). In combination with our main results, this suggests that when playing with digital novel toys, older children in our studies were both more variable in their exploration and could better recall information on how the toys worked. However, the current work is limited to this correlational finding, and additional work is warranted to confirm causal links among development, exploration, and learning.

## 4 Discussion

We began this investigation with the goal of better characterizing children's exploration in naturalistic environments. We specifically focused on the developmental changes in exploratory behavior, with a focus on how play may reveal exploratory tendencies that are not inherently anchored in information gain and reward, but instead in specific preferences to persevere in one's current investigation or adaptively shift focus. We approached this question with computational modeling, extending well‐known methods in decision making—Markov Chain decision models—and applying them toward the goal of better understanding exploratory play.

Overall, we found that, as children get older, they explore novel environments more variably, shifting between potential affordances with a higher tendency. We also found that, as children get older, they are less likely to perseverate on a single affordance. Finally, we found that, across tasks, older children are more likely to end exploratory play earlier than younger children. These results replicate main findings from Pelz and Kidd (2020) who found that as children get older, their exploratory actions in a tablet game reflect more complexity; this complexity arose because of more variability in exploratory patterns, or because of less perseveration, or some combination of both.

In follow‐up task‐by‐task analysis, our analysis revealed that our main results were primarily driven by exploratory behaviors in the Novel Toy tasks, rather than the Envelope and Science Facts task. The Envelope task had a more restricted age range, and both Envelope and Science Facts tasks had fewer observations, and thus less power. Nonetheless, trends were quite flat in both these tasks, and in some cases even counter to the overall results driven by the Novel Toy data, suggesting they promoted different behavioral strategies for children.

The three tasks differed from each other in several important ways. The Science Facts task was perhaps the least “toy‐like”; children were simply clicking on items to gather a single fact about the outcome. As a result, exploration was likely primarily driven by goal‐directed information‐seeking alone. Other intrinsic rewards associated with exploring toys—including observing surprising and stimulating outcomes and having some agency over causing those effects—were not present in the Science Facts task. While the Envelopes task included opportunities for play, children's actions were primarily driven by initial exploration of the envelopes—to open and reveal the hidden contents inside. In this way, the task more closely mirrored the Science Facts task than the online Novel Toy simulator. These results reveal the importance of taking individual task structures into account when making broader claims about exploratory behaviors. They also raise questions regarding how developmental differences in inferring task goals, information gain, and expectations around reward structures might differently drive exploration.

As a follow‐up exploratory analysis, by accidental virtue of the original experimental methods, we were able to investigate the learning outcomes following children's playful exploration in the Novel Toy datasets. Our results revealed that older children were more likely to answer the learning check questions correctly. This suggests that with age, children's exploratory play better supported independent discovery.

That in our task older children were better learners also dovetails with the explanatory account suggested by Pelz and Kidd (2020) and Bass and Bonawitz (2024). Pelz and Kidd (2020) suggest that the developmental changes in children's playful exploration in their naturalistic learning environments is a reflection of the emergence of greater “efficiency” with age. Such efficiency may stem from differences in cognitive capacity. Younger children's playful choices could still reflect rational decision making: for example, if younger children have challenges with memory and encoding, then greater perseveration could be an adaptive strategy for retaining information following playful interventions. Greater exploratory efficiency may also relate to choosing actions more effectively to optimize information gain from the environment, aligning with the concept of “directed exploration.” Whatever the case, these results do not provide evidence that in novel exploratory play tasks younger children's choices are more “random” than older children's. If so, we would have expected more variability as marked by higher switching scores in *younger* children, contrary to our results. This raises important questions about how to interpret differences between the outcomes found here and the work finding greater variability in younger children's exploratory behaviors.

A second difference between these tasks is in the operationalization of the term “breadth.” In the Pelz and Kidd (2020) tasks, variability is reported, as aligning with our measure of “switching” here. However, in other work, breadth is often characterized as the number of unique actions attempted. Children could have repeatedly switched between just a few different aspects of the toys, leading to a high switch rate, but not reflecting breadth in the “total unique actions” sense. This is a potentially important difference and one that warrants more attention. Thus, we also coded our data to include a measure of this more traditional “breadth” score. The Envelopes Task and the Science Facts tasks do not have a meaningful measure of breadth, because every science fact is new (and thus total facts explored is the same as breadth) and because in the Envelopes Task, essentially all children opened all envelopes. We did look at this score for the Novel Toys task, however. Our Markov Model cannot be used to explore this finding, but running a mixed effects regression, with age and condition as the predictors and breadth of exploration as the outcome, we find a significant positive relationship between age and outcome (*β* = 0.97, *SE* = 0.22, *t* = 4.384, *p* < 0.001); older children showed greater breadth in exploration, providing converging results with switching in our tasks.

Our findings on children's exploratory play also align conceptually with the literature on children's categorization in the sequential touching literature. Previous work has modeled the action‐by‐action behaviors of children's category learning using Mixture models and Monte Carlo simulations (Mandler et al. 1991; Mareschal and Tan 2007, 2008). These methods may be considered more complex and use optimization methods to capture the underlying behavioral patterns that drove children's touch behaviors during the tasks. In contrast, our simpler Markov chain model's estimates were deterministic and described children's behaviors across diverse play settings. Together, our work and the sequential touching literature demonstrate the value of highlighting the generalizability of computational modeling techniques for sequences of children's behaviors in play, less‐structured contexts. Here, our current method is uniformly applied across diverse play environments and task structures. We leveraged this flexibility to systematically compare exploratory behavior across five unique studies involving three distinct types of tasks, demonstrating the broader applicability of such frameworks for developmental research. While this generalizable nature of our approach is a key contribution of the current work, the differing task structures may underlie the apparent discrepancies found across studies.

### 4.1 What Might Explain Empirical Discrepancies of Children's Exploratory Play?

Numerous differences between tasks could account for the apparent tension between early‐breadth and late‐breadth findings. We briefly consider whether the complexity of the search space and children's prior beliefs about the space can help resolve this tension. In particular, we suggest that understanding the difference between directed and random exploration, and the conditions under which these are deployed, can help reconcile results that seemingly differ regarding the development of exploratory breadth.

At least three competing factors underlie most search tasks: exploitation, random exploration, and directed exploration (Gershman 2018; Wilson et al. 2014, 2021; Meder et al. 2021). To exploit rewards in these environments, agents must optimize search strategies to quickly learn about the reward landscape. Learners could explore noisily, testing ideas without clear goals or “optimality,” reflecting greater randomness in their decisions. Alternatively, as in directed exploration, learners seek to minimize uncertainty (Gottlieb and Oudeyer 2018), deploying expectations about the environment to search in locations that will maximize information gain.

Expectations about the environment and its likely affordances will drive different exploratory patterns. A potential explanation for why some tasks reveal conflicting findings to those here and seem to elicit broader search by younger children is that the prior beliefs pertaining to the task demands differ. In many domains, children have fewer inductive biases constraining the space of options they consider (Gopnik et al. 2015). In tasks that involve mentally searching in an undefined hypothesis space to hypothesis test, children may be less efficient explorers in that they will spend time testing adult‐deemed “unlikely” hypotheses; these same hypotheses won't be explored by older children and adults who reasonably “rule them out” as potential opportunities to reveal meaningful evidence a priori. However, as a result, in cases when there is a “learning trap,” such that an unlikely or uncommon hypothesis explains data, younger children may be more likely to discover it (e.g., Liquin and Gopnik 2022; Lucas et al. 2014). Without strong inductive constraints guiding mental exploration, younger children's hypothesis search will appear more broad. Note that, if tasks are deployed in domains where children have more uniform priors, exploratory breadth would be present regardless of whether children are carrying‐out directed or random exploration strategies. That is, even if engaging in directed exploration, if an agent's beliefs about what hypotheses are likely true are more uniform, then the information gain for exploring any particular action is also more uniform. Optimizing directed exploration over a more uniform space will result in more variable exploration as compared to optimizing directed exploration over a less uniform space.

In contrast, in our (and arguably Pelz and Kidd's (2020), and Bass and Bonawitz's (2024)) Novel Toy paradigm, the utility over the space of possibilities (e.g., which food an animated monster will like; which physical features likely hide causal affordances) was likely more uniform across development (younger and older learners likely have similar beliefs about what foods or functions exists in these paradigms). As a result, there would be no reason to expect broader exploration in earlier development on these tasks if children were exploring in a directed fashion. But this raises the question of why younger children were not more *random* explorers in our tasks, as compared to other work suggesting they are.

This brings a second explanation to bear on why we observe differing evidence around developmental shifts in exploratory variability: whether children are *able* to perform directed exploration or whether the complexity of the task structures results in more random exploration. Accounts that tend to find evidence for random exploration driving breadth involve spaces that are larger, difficult to search over, and require maintaining expectations about non‐uniform utilities. For example, tasks designed to maximize reward, as in Meder et al.’ (2021) explore‐exploit tasks, often involve uncovering rewards in a physical space that cannot be fully explored, while simultaneously needing to integrate the expectation of information over more complex prior landscapes. These explore‐exploit tasks tend to find younger children defaulting to more “random” exploration; this may be a resource rational strategy (Lieder and Griffiths 2019) when the computational demands of computing optimal information gain for direct exploration are high. In contrast to explore‐exploit studies like Meder et al. (2021), our Novel Toy task and Pelz and Kidd's (2020) monster food task both provide clear visual constraints on possible toy functions or food elements to test. This may support directed exploration by providing a more explicit and intuitive approach to testing items. In sum, tasks that involve simpler expected‐value computations may reveal that directed exploration emerges with age, which may appear more exploratorily variable in landscapes with relatively uniform expectations on outcomes.

### 4.2 Limitations and Future Work

The current work provides initial insights into developmental changes in children's exploratory play–but several limitations must still be acknowledged. First, the generalizability of our findings may not extend to other cultural contexts, with the majority of our sample being westernized regions. Study 2 was conducted in the United Kingdom, but all other studies were performed with children in the United States. Cross‐cultural research shows that cultural factors influence children's learning strategies and exploration (Shneidman et al. 2016). As proposed in past work, children from more stressful backgrounds may adapt their exploration in meaningful ways not captured here (Bass and Bonawitz 2024; Frankenhuis and Gopnik 2023; Tooley et al. 2021). Investigating the interactions between socioeconomic factors, environmental context, and developmental changes may highlight additional factors that lead to developmental changes in behavioral patterns of exploratory play. Future work should examine whether the developmental trends and contextual factors identified here hold across diverse populations.

The work presented here provides an analytical approach to describing developmental shifts in children's exploratory play. However, an important next step is to provide explanatory accounts of the specific processes underlying play. The models employed in the current work are descriptive (as characterized by Luce 1995), and our findings lend themselves to a structured overview of the developmental trends during children's play but do not substantiate any causal conclusions on their own. We have provided some explanatory accounts for our findings above, but future computational models of cognition could test this explanatory framework in carefully controlled settings. For example, we are inspired by computational cognitive development work that has begun to examine the explanatory factors underlying playful exploration (e.g., Du et al. 2023; Lidayan et al. 2025). Future work is needed as we refine our theoretical accounts of developmental transitions in exploration.

## 5 Conclusion

Characterizing the complex development of children's playful exploration requires an interdisciplinary bridge that can account for both the qualitatively‐unique contexts play presents, while extending quantitative computational models that typically focus on reward and information gain. We leveraged Markov models as a computational approach to analyze exploratory play across varied tasks and to investigate broad developmental shifts in children's naturalistic play. Our results suggested that in Novel Toy exploration tasks, in particular, children may be broader explorers as they age. We provided an explanatory account for the apparent discrepancy between past findings, suggesting that task‐specific details such as the distribution of expected information and reward, the developmental shifts of children's prior beliefs, and the complexity of the task environment could be the differing underlying factors that lead to this incongruity. Rather than perseverating on our results here, however, we suggest that broader exploration will be needed to uncover the explanatory principles at play.

## Ethics Statement

The methods of all studies reported here were approved by their investigators' respective Institutional Review Board (IRB) at the time of data collection. All participating families provided informed consent prior to participating.

## Conflicts of Interest

The authors of this submission, listed above, certify that they have no affiliations with or involvement in any organization or entity with any potential sources of conflict of interest, whether they be of financial or non‐financial interest in the subject matter or materials discussed in this manuscript.

## Supporting information




**Supporting File 1**: desc70081‐sup‐0001‐appendix.docx

## Data Availability

Anonymized versions of the data and code that support the findings of this study will be available via the Open Science Framework at https://osf.io/agyju/?view_only=62c27a0372474e93a0f5a7c05ceaafb5.

## Endnotes

1 Pelz and Kidd control for overall number of actions (length of play) in their models, but as a result, it is unclear from the analysis whether this factor is also changing with development.

2 Preparation and implementation of these Markov models were conducted in Python using Jupyter Notebook for interactivity (Kluyver et al. 2016).

3 The analyses described here were conducted primarily using Python in Jupyter Notebook (Kluyver et al. 2016), with RPy2 (Gautier 2008) and Pymer4 (Jolly 2018) that allows for us of specific statistical analyses in R (R Core Team 2019).

4 For example, the Zero‐order correlations among these scores were significant: with a negative correlation between stay and switch (*r*(660) = –0.979, *p* < 0.001), a negative correlation between stay and end (*r*(660) = –0.207, *p* < 0.001), but no significance between switch and end (*r*(660) = 0.004, *p* = 0.93).

5 The scores as‐is and min–max normalized scores led to identical correlations within studies, so the correlations with age within study will consider the original play transition scores.
