# Supplementary material for: Computational Approaches Reveal Developmental Shifts in Exploratory Play
Source: Dev Sci. 2025 Oct 25;28(6):e70081. doi: 10.1111/desc.70081 (PMC12553326; doi:10.1111/desc.70081)
Supplement: Supplementary file 1 — Supporting File 1: desc70081‐sup‐0001‐appendix.docx [file DESC-28-e70081-s001.docx]

# Appendix

Visual depictions of the different types of exploratory play tasks children participating in across the five studies (Figure 1A for Studies 1 and 2, Figure 2A for Studies 3 and 3, Figure 3A for Study 5).

| 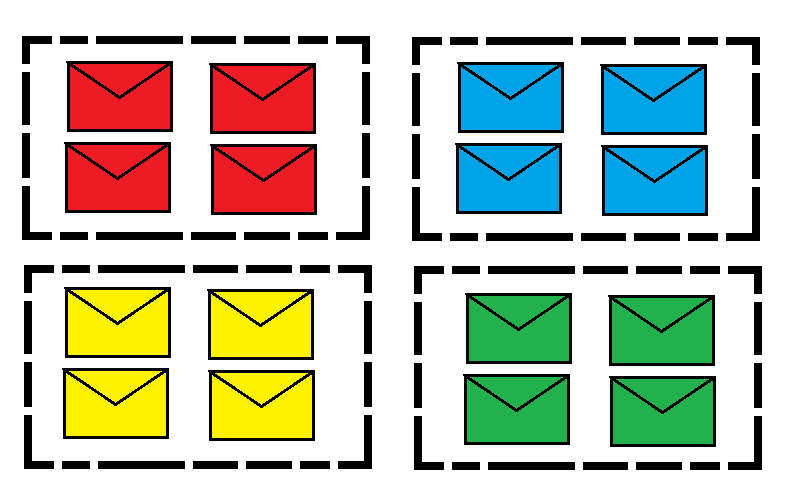 |
| --- |
| Figure 1A. In Study 1 and Study 2, children received a package with 16 envelopes and an instruction sheet for parents that requested their help setting up a play area. Four themed, differently colored sets of four envelopes were laid out in a grid-like fashion as shown above. Children were then prompted with a pedagogical question (“How might the things in the [target color] envelopes go together? Can you think about that?”) before being allowed to play. Children’s play was recorded, with secondary coders listing the sequence of colors in which envelopes were opened. Here, exploration was considered as the “color-by-color” sequence that children opened envelopes in, as well as the sequence of play with the item already opened (but labeled according to the original color-category of the item given its original envelope color). This provided a measure of whether sequential play choices were within color-category or switching to a new color category. |

| 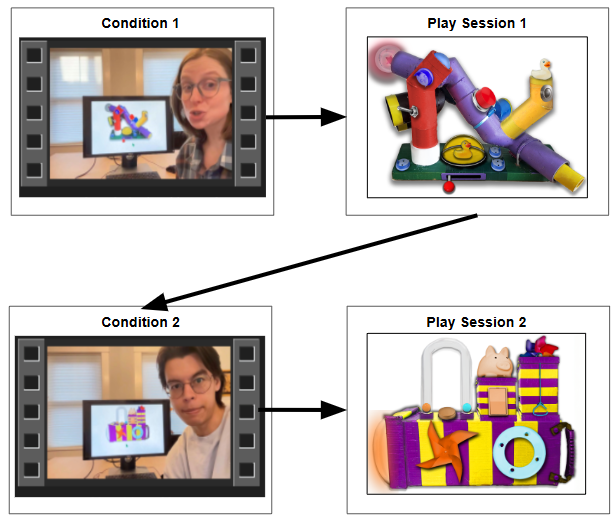 |
| --- |
| Figure 2A. In Study 3 and Study 4, children first saw one of two teachers demonstrate a specific function on a virtual Novel Toy on a computer screen. Both conditions had a demonstration, but varied on whether the demonstration was done intentionally (e.g., “This is my toy! I’m going to show you how my toy works …”) or was performed accidentally (e.g., “I’ve never seen this toy before! I wonder how it works… Oops, did you see that?”). After watching this first video, the child was then allowed to play with the digital toy in the video. The digital toy contained many possible visual affordances that could link to possible outcomes (e.g. semi-surreptitious buttons that made fans spin, lights turn on, music play, revealed hidden compartments, etc). After confirming they were done playing, or after their allotted time expired, children then saw a second, new teacher’s video in the remaining condition before being given the opportunity to play with a second, different Novel Toy. Children’s click-by-click behavior (for both active and inert regions of the toys) was automatically recorded to track their exploratory decisions such as activating or turning off the same functions repeatedly or testing other potential functions. This provided a measure of whether sequential play choices were within persisted on a single affordance or shift among multiple parts of the toys. |

| 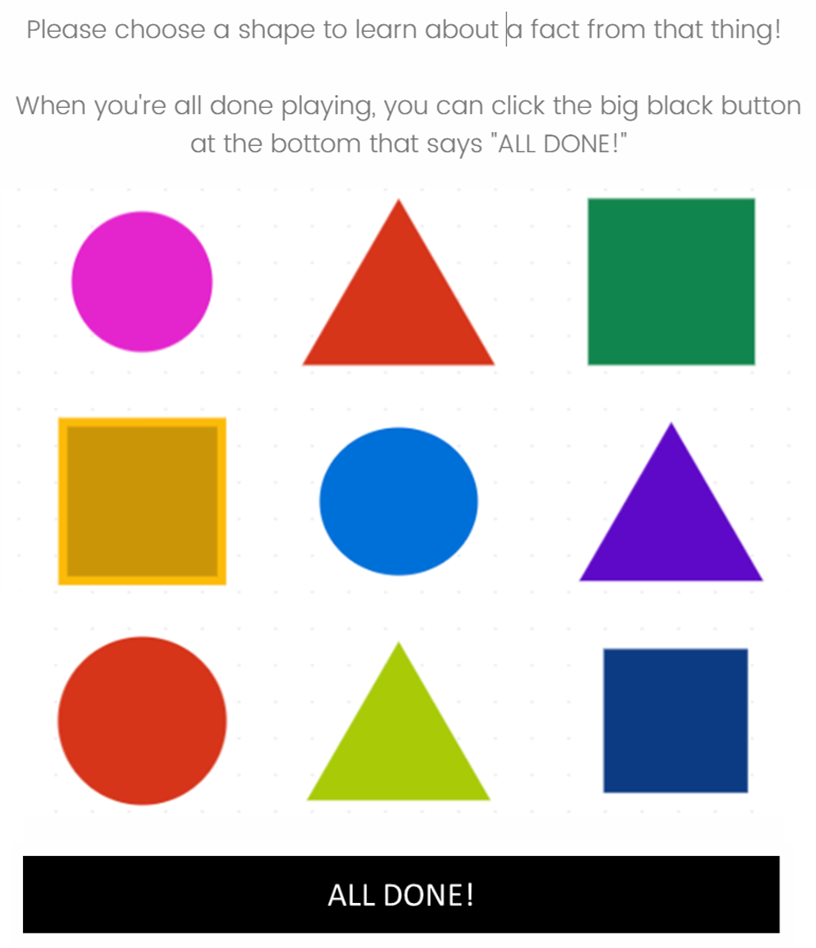 |
| --- |
| Figure 3A. Design of the Science Facts task in Study 5. The task is a modified version of the “Explore and Learn” task in Evans & Jirout (2023). A grid of nine shapes were shown on the child's screen. Each shape was matched to a specific topic, and each topic had five unique facts. Clicking on any of the shapes transitions to a page with a “cool fact” about the topic, narration of the fact, and accompanying picture. After exhausting all five facts within a topic, the sixth click (and all clicks afterward) made a new sixth screen pop up with text and a narration stating “Wow! You got to hear all of the facts that we have about [*topic*]! If you'd like, you can go back to the previous page and pick another topic to learn about.” Children’s exploration ended when they clicked on “ALL DONE!” and then clicked on the “YES” button of a confirmation page (“Are you all done playing?”). Children’s exploration was measured by tracking the click-by-click transition among the available topics, allowing us to measure whether children explored one topic deeply, or switched among different topics. |

#

#

| Table 1A. Follow-up, exploratory analysis within task type (Themed Envelopes, online Novel Toys, or Science facts) of the partial correlations between age and children’s transition scores (stay, switch) while controlling for end scores. Significant correlations are noted with and asterisk (*) and bold formatting. The partial correlation results replicate the reported correlations within-study, where significant relations between age and both play scores are only found for play with Novel Toys (Middle Row, Studies 3 and 4). Again, partial correlations were not found in either of the Themed Envelopes (Top Row; Studies 1 and 2) or Science Facts paradigms (Bottom Row; Study 5). This exploratory, but conservative analysis suggests that in specific contexts, such as in play with Novel Toys, children’s exploratory play may shift from a preference for persistence toward more variable exploratory choices, despite possible individual differences for the length of their play session. | | |
| --- | --- | --- |
| Task Used | Partial Correlation between Age & Score  Controlling for End Scores | |
|  | *Stay  Score* | *Switch  Score* |
| Themed Envelopes  (Study 1 & Study 2) | *r*(161) = 0.0342  CI 95% [-0.12, 0.19]  *p* = 0.665 | *r*(161) = -0.0342  CI 95% [-0.19, 0.12]  *p* = 0.665 |
| Novel Toy  (Study 3 & Study 4) | ***r*(426) = -0.289**  **CI 95% [-0.37, -0.2]**  ***p* < 0.001*** | ***r*(426) = 0.289**  **CI 95% [0.2, 0.37]**  ***p* < 0.001*** |
| Science Facts  (Study 5) | *r*(161) = -0.126  CI 95% [-0.11, 0.35]  *p* = 0.297 | *r*(161) = 0.126  CI 95% [-.35, 0.11]  *p* = 0.297 |

#

***Analysis of Children’s First Play Sessions***

To ensure that our results were not unduly influenced by potential non-independence of repeated measures within children, we conducted additional analyses using just the first play session from each child. For this “first-play” subset, we found that the main effect remains even when only including children’s first play sessions (432 of the original 662 observations). First, the correlation results remained the same, where we found a negative correlation between age and children’s stay scores (r(430) = -0.197, p < 0.0001); a positive correlation between age and switch scores (r(430) = 0.172, p < 0.001); and a positive correlation between age and children’s end scores (r(430) = 0.133, p < 0.01). Second, we looked at the partial correlations between age and both stay or switch scores, while controlling for end scores. We again found that children’s age had a significant negative correlation with their stay scores (r(432) = -0.173, CI 95% [-0.26, -0.08], p < 0.001), and a significant positive correlation with their switch scores (r(432) = 0.163, CI 95% [0.08, 0.26], p < 0.001). Third, we reran the Dirichlet regression model to assess the relative transition probabilities as a function of age. Here, we treated stay scores as the reference category to interpret the effect of age on switching play targets and ending play. Here, the regression results for this “first-play” subset again indicated that age significantly predicted children’s play transitions, where age was positively related to both switch (β = 0.12597, SE = 0.02305, z = 5.462, p < 0.0001) and end scores (β = 0.1090, SE = 0.0330, z = 3.303, p < 0.001). Overall, our results remain for this “first-play” subset within the dataset (n = 432 of 662 original observations), suggesting our findings are not solely driven by multiple observations from the same individuals, and appear to hold when considering only one session per child.

# 
